# Curcumin derived from medicinal homologous foods: its main signals in immunoregulation of oxidative stress, inflammation, and apoptosis

**DOI:** 10.3389/fimmu.2023.1233652

**Published:** 2023-07-11

**Authors:** Ping Hu, Kaiqi Li, Xiao-Xu Peng, Yufei Kan, Tong-Jia Yao, Zi-Yu Wang, Zhaojian Li, Hao-Yu Liu, Demin Cai

**Affiliations:** ^1^ College of Animal Science and Technology, Yangzhou University, Yangzhou, China; ^2^ International Joint Research Laboratory in Universities of Jiangsu Province of China for Domestic Animal Germplasm Resources and Genetic Improvement, Yangzhou, China

**Keywords:** curcumin, oxidative stress, inflammation, apoptosis, immuno-regulation

## Abstract

It has been for thousands of years in China known medicinal homologous foods that can be employed both as foods and medicines to benefit human and animal health. These edible herbal materials perform divert roles in the regulation of metabolic disorders, cancers, and immune-related diseases. Curcumin, the primary component derived from medicinal homologous foods like curcuma longa rhizome, is reported to play vital actions in organic activities, such as the numerous pharmacological functions including anti-oxidative stress, anti-inflammation and anti/pro-apoptosis in treating various diseases. However, the potential mechanisms of curcumin-derived modulation still need to be developed and attract more attention worldwide. Given that these signal pathways are enrolled in important bioactive reactions, we collected curcumin’s last achievements predominantly on the immune-regulation signals with the underlying targetable strategies in the last 10 years. This mini-review will be helpful to accelerate curcumin and other extracts from medicinal homologous foods use in future human clinical applications.

## Introduction

Oxidative stress, inflammation, and apoptosis are interrelated processes that play pivotal roles in a variety of physiological and pathological conditions (1). Oxidative stress denotes an imbalance between reactive oxygen species (ROS) production and the body’s antioxidant defense mechanisms, resulting in cellular dysfunction and damage (2), while inflammation is an immune system response to harmful stimuli, such as tissue injury or pathogens, characterized by the recruitment of immune cells and the release of inflammatory mediators (3). Oxidative stress has been proven to activate pro-inflammatory signaling pathways and produce inflammatory cytokines and chemokines, thereby triggering inflammation (4). In turn, inflammation can increase oxidative stress by promoting ROS production and disrupting antioxidant defense mechanisms. Additionally, oxidative stress and inflammation can activate pro-apoptotic signaling pathways and inhibit anti-apoptotic pathways, ultimately leading to cell death (5, 6). Apoptosis, also known as programmed cell death, is a tightly controlled mechanism of cellular demise that serves a vital role in maintaining tissue homeostasis and eliminating damaged or abnormal cells (7, 8). The relationship among oxidative stress, inflammation, and apoptosis is multifaceted and complex. Dysregulation of these processes can contribute to the development and progression of various diseases, including metabolic disorders, immune-related diseases, and cancers (9–11). Therefore, it is crucial to comprehend the mechanisms underlying these processes and identify potential therapeutic targets to regulate them in preventing and treating these diseases.

The close interplay between oxidative stress, inflammation, and apoptosis has led to the investigation of medicinal homologous foods with these properties. Medicinal homologous foods epitomize a remarkable amalgamation of food and medicine. In addition to their inherent nutritional value, these foods harbor extracts imbued with distinct properties that contribute to disease prevention, treatment, and a range of healthcare benefits (12). Derived from natural sources, medicinal homologous food extracts encompass bioactive compounds with notable therapeutic potential, including polyphenols, flavonoids, terpenes, and alkaloids (13). These compounds synergistically contribute to the medicinal attributes of these foods. Curcumin (CUR) is the primary active compound found in turmeric, constituting approximately 8% of most turmeric preparations derived from curcuma longa (14). It serves as a widely used flavoring agent in food supplements and is responsible for imparting the characteristic yellow color to turmeric spice. Due to its role in oxidative stress, inflammatory response, and apoptosis, CUR shows significant pharmacological potential and has exhibited favorable effects concerning various metabolic disorders, immune-related diseases, and cancers (15, 16). Growing experimental evidence revealed that CUR had been shown to scavenge ROS, inhibit the production of pro-inflammatory cytokines, and modulate various signaling pathways involved in apoptosis (17, 18). Furthermore, CUR exhibits an excellent safety profile, with no significant adverse effects reported even at high doses (19). This feature makes it an attractive candidate for therapeutic interventions, either alone or in combination with other drugs, to enhance treatment outcomes and minimize side effects. Therefore, the investigation of CUR’s potential use in the treatment of oxidative stress, inflammation, and apoptosis can provide valuable theoretical strategies for developing it as a promising candidate for the prevention and treatment of various diseases.

## The anti and pro-oxidant properties of Curcumin

Oxidative stress (OS) is a pathological state that results from an imbalance between the pro-oxidant and the antioxidant species (20). OS induces several oxidative intermediates and promotes protease secretion, and neutrophilic inflammatory infiltration, ultimately resulting in cellular senescence and various diseases, including cancer, cardiovascular disease, and neurodegenerative disorders (21, 22). Reactive oxygen species (ROS) are by-products produced during oxygen metabolism, comprising free radicals and non-free radicals that can cause oxidative damage to cellular components, including lipids, proteins, and DNA, which can disrupt cellular signaling pathways (23). As a protective mechanism, cellular antioxidant defense systems, including SOD (superoxide dismutase), peroxidases, antioxidants, and vitamins, have evolved as a protective mechanism to maintain the oxidation-reduction (redox) status by preventing the accumulation of ROS (24).

CUR is a naturally occurring compound derived from plants that exhibit anti-oxidative stress properties. Research studies have reported that CUR effectively attenuates the release of ROS in various experimental settings, including cell lines, preclinical models, and clinical samples (25–27). Pretreatment with CUR regulates the expression of antioxidant enzymes through nuclear factor erythroid 2-related factor 2 (Nrf2) signaling pathways to stabilize ROS levels (28). Nrf2 is a transcription factor that plays a critical role in the cellular response to oxidative stress by regulating the expression of genes that encode antioxidant enzymes and detoxifying proteins (29). Under normal conditions, Nrf2 is inhibited by Kelch-like ECH-associated protein 1 (Keap1), which targets Nrf2 for ubiquitination and proteasomal degradation. In response to oxidative stress conditions, specific cysteine residues within the Keap1 protein undergo oxidation, including Cys-151, Cys-273, Cys-288, Cys-297, and Cys-257, leading to the dissociation of the NRF2-Keap1 complex (30). A study conducted on the therapeutic potential of CUR in mouse skin revealed the binding of CUR to Keap1 Cys151 (31). This finding suggests that the modification of this specific amino acid by CUR may play a crucial role in releasing NRF2 from Keap1, thus highlighting its potential as a therapeutic target (32). Upon activation, Nrf2 translocates to the nucleus, binding to antioxidant response elements (AREs) and promoting the expression of ARE genes (33). The dissociation of NRF2 from the NRF2-Keap1 complex is an essential step in activating the NRF2/ARE signaling pathway, which increases the expression of antioxidant enzymes, including glutathione peroxidase (GPx), superoxide dismutase (SOD), and catalase (CAT), as well as phase II antioxidant enzymes such as heme oxygenase-1(HO-1) and NAD(P)H quinone dehydrogenase 1 (NQO1). These enzymes eliminate ROS and maintain redox homeostasis (34, 35). Studies have found that CUR enhances the activities of antioxidant enzymes through the Nrf2 pathway, which contributes to neutralizing ROS and protecting the host from damage caused by oxidative stress (36, 37).

A recent study has indicated that CUR can alleviate intestinal barrier injury and mitochondrial damage induced by oxidative stress through the activation of AMP-activated protein kinase (AMPK) pathway (38). AMPK is an important regulator of cellular energy homeostasis and is activated in response to low energy status. In addition to its role in energy metabolism, AMPK has been demonstrated to play a crucial role in the regulation of cellular stress response pathways, including the Nrf2 pathway (39). Phosphorylation of Nrf2 by AMPK results in its translocation to the nucleus and activation of Nrf2-dependent gene expression. AMPK activates Nrf2 through direct phosphorylation at Ser550, leading to the nuclear accumulation of Nrf2 (40). This phosphorylation enhances Nrf2 transcriptional activity and promotes the expression of Nrf2 target genes, such as SOD-1 and HO-1, thereby attenuating oxidative stress and inflammation (41). Therefore, the activation of AMPK enhances antioxidant defenses and provides cellular protection against oxidative stress but also represents an important mechanism for CUR to maintain cellular redox homeostasis and protect cells from oxidative stress by activating Nrf2 (42).. It is worth mentioning that CUR initiates a pro-oxidant response that stimulates the ROS and lipid MDA into less harmful products by inhibiting reduced glutathione (RGS) and GPx, which enhances cell death in cancer cells (43). Moreover, growing evidences confirm the effective suppression of gastric cancer cell proliferation by CUR through the induction of ROS generation, subsequently triggering apoptosis (44, 45). Overall, a comprehensive understanding of the intricate details of anti/pro-oxidative properties of CUR could pave the way for the development of CUR strategies aimed at preventing and treating oxidative stress-related diseases.

### The anti-inflammatory property of Curcumin

Inflammation is a complex biological response initiated by diverse stimuli, including pathogens, tissue damage, and cellular stress. In response to these stimuli, immune and other cells produce a diverse group of molecules known as inflammatory mediators, including cytokines, chemokines, prostaglandins, and leukotrienes (46). While inflammation and inflammatory mediators are essential for the immune response and play a role in fighting infections and promoting tissue repair, excessive or chronic inflammation may develop various diseases, such as autoimmune disorders, metabolic disorders, and cancer (47–49). Therefore, modulation of inflammatory mediators has the potential to be a promising strategy for preventing and treating these conditions.

The efficacy of CUR in modulating the inflammatory response can be attributed to its capability to regulate multiple signaling pathways and molecules involved in inflammation. Numerous studies have demonstrated that CUR exerts anti-inflammatory effects by inhibiting the activity of several pro-inflammatory mediators, including cytokines (e.g., TNF-α, IL-1β, IL-6), chemokines (e.g., CXCL8, CCL2), and enzymes (e.g., COX-2, iNOS) (50, 51). The regulation of the inflammatory response by CUR is attributed to its ability to block the NF-κB (nuclear factor kappa-light-chain-enhancer of activated B cells) pathway, which is vital in the inflammatory process (52). NF-κB, a ubiquitously present transcription factor, critically regulates the immune response, inflammation, cell proliferation, and apoptosis while participating in the expression of genes that regulate cellular responses to various stimuli, including stress, cytokines, free radicals, and microbial pathogens (53). In an inactive state, NF-κB is bound to inhibitor proteins, which prevent it from entering the nucleus and activating gene transcription. Upon activation, NF-κB translocates to the nucleus and activates the transcription of various pro-inflammatory genes. Moreover, CUR administration efficiently blocks the phosphorylation of the inhibitor of kappa B (IKK) and the inhibitor of NF-κB (IκB), which are necessary for the translocation of NF-κB into the nucleus in ulcerative colitis and cancer cells (54, 55). Furthermore, CUR also suppresses the activation of Toll-like receptors (TLRs) and downstream signaling pathways that lead to NF-κB activation (56). Overall, these findings indicate that CUR inhibits TLR activation and blocks the phosphorylation of IKK and IκB, subsequently preventing the translocation of NF-κB towards the nucleus. Ultimately, this cascade of events effectively inhibits the transcription of various pro-inflammatory genes.

The Activator Protein-1 (AP-1) pathway is an important pathway involved in CUR’s regulation of the inflammatory response (57). AP-1 is a transcription factor that promotes the transcription of several pro-inflammatory genes, such as cytokines, chemokines, and matrix metalloproteinases (MMPs) (58, 59). In line with this, Woo et al. revealed that CUR blocks AP-1 activation by inhibiting JNK, which phosphorylates and activates c-Jun (an AP-1 subunit) (60). Moreover, it has been demonstrated that CUR can directly bind to c-Jun and inhibit its DNA binding activity, thereby suppressing the expression of pro-inflammatory genes and alleviating inflammation (60). Furthermore, CUR can also depress the activity of inflammation-related enzymes (e.g., COX-2, iNOS) by inhibiting p38 MAPK signaling (61). Aside from its impact on inflammatory mediators, CUR has been observed to modulate the activity of immune cells involved in the inflammatory response, suppressing the production of pro-inflammatory cytokines TNF-α and IL-6 by macrophages and inducing the polarization of macrophages towards an anti-inflammatory M2 phenotype (62). These studies provide insights into the regulation of signaling pathways by CUR, which has been shown to inhibit the activation of NF-κB, AP-1, and MAPK, as well as the expression and activity of numerous pro-inflammatory mediators, underscoring its potential as a therapeutic agent for various inflammatory disorders.

### The anti and pro-apoptosis properties of Curcumin

Apoptosis, also referred to as programmed cell death, is a precisely regulated process that eliminates impaired or unneeded cells to maintain tissue homeostasis (63). Disruption of apoptosis can result in various pathological conditions, including autoimmune diseases, cancer, and neurodegeneration (11, 64, 65). Apoptosis is governed by an intricate network of signaling pathways, which include extrinsic and intrinsic pathways (66). The extrinsic pathway is triggered by the binding of extracellular ligands to death receptors (e.g., TNF, Fas receptors). In contrast, the intrinsic pathway is initiated by intracellular stress signals, such as oxidative stress and inflammatory response (67, 68). Therefore, comprehending the molecular mechanisms that regulate apoptosis and identifying effective strategies for modulating apoptosis is critical for preventing and treating diseases.

Compelling scientific and dietary evidence has demonstrated the potential of CUR to induce apoptosis in various cancer cells. For instance, CUR has been extensively investigated for its potential to inhibit the PI3K/AKT pathway in cancer cells, leading to the downregulation of downstream targets such as mTOR and inducing apoptosis (69, 70). The activation of the PI3K/AKT pathway, triggered by the production of 3’-phosphorylated phosphoinositides, is a critical signaling pathway involved in both apoptosis and cell cycle progression (71). Abnormal activation of the PI3K/AKT pathway hinders apoptosis by upregulating anti-apoptotic genes such as Bcl-2 and downregulating pro-apoptotic genes like Bax (71). In addition, CUR is validated to upregulate the expression of PTEN, a tumor suppressor that negatively regulates PI3K/AKT signaling, while downregulating the expression of genes involved in AKT activation (72). Moreover, CUR modulates upstream regulators of the PI3K/AKT/mTOR pathway, including growth factor receptors and integrins. Chiu et al. reported that CUR inhibits the activity of EGFR, a receptor tyrosine kinase that activates the PI3K/AKT pathway, leading to apoptosis induction in cancer cells (73). Aside from PI3K/AKT/mTOR pathway, the Janus kinase/signal transducer and activator of transcription (JAK/STAT) pathway is another major signaling pathway that regulates apoptosis (74). The JAK/STAT pathway is a signaling cascade that plays a critical role in the regulation of cell proliferation, differentiation, and apoptosis, and its dysregulation has been implicated in various diseases (74). In a myeloproliferative neoplasms model, CUR activates the JAK2/STAT pathway, inducing apoptosis and inhibiting proliferation, thus exerting an antitumor effect on human JAK2-mutated cells (75). Furthermore, it has been noted that CUR modulates several apoptotic pathways, including the death receptor and endoplasmic reticulum (ER) stress-induced apoptosis pathways (76). The death receptor pathway is initiated when death ligands, such as TNF and Fas ligand, bind to their respective receptors, activating caspase-8 and downstream effector caspases, ultimately leading to apoptosis (77). CUR has been shown to sensitize cancer cells to death receptor-mediated apoptosis by promoting Fas-receptor and by activation of caspase-3 and -8 in tumor cells (78). In addition, CUR activates the ER stress-induced apoptosis pathway, a critical membranous organelle during apoptosis, and participates in complex interaction with the mitochondria (79). Furthermore, in tumor therapy, CUR induces ER stress. It activates the unfolded protein response (UPR), leading to the upregulation of the pro-apoptotic proteins (CHOP and Bax) and the activation of caspase-3, which ultimately triggers apoptosis and exerts anti-tumor property (80). Experimental data have conclusively proved that CUR exerts anti-cancer effects by promoting apoptosis through several signaling pathways in cancer cells.

Intriguingly, CUR has also been demonstrated to possess inhibitory effects on apoptosis in non-cancerous diseases, expanding its potential therapeutic applications beyond cancer treatment. In diabetic cardiomyopathy, CUR inhibits apoptosis and alleviates oxidative stress by eliminating ROS levels and activating PI3K-AKT signaling pathways, resulting in the downregulation of caspase-3 and Bax protein expression (81). Moreover, CUR treatment was shown to attenuate apoptosis and inflammation by inhibiting JAK2/STAT3 and NF-κB signaling pathways, which results in the upregulation of Bcl-2, and the downregulation of Bax and caspase-3 in an acute kidney injury model (82). In addition, the treatment of CUR enhances the viability of Saos-2 cells, mitigates apoptosis, improves mitochondrial membrane function and potential, and upregulates the phosphorylation of GSK3b and protein kinase B (AKT) (83). Furthermore, in the gentamicin-induced nephrotoxicity model, CUR reverses the expression levels of ER stress markers (CHOP, calpain-2, caspase-12, and cleaved caspase-7) and decreased apoptotic protein biomarkers expression (cleaved caspase-3 and Bax) (84). Therefore, besides its anticancer effects through promoting apoptosis in cancer cells, CUR has also demonstrated the ability to inhibit apoptosis in non-cancerous diseases, such as diabetic cardiomyopathy and acute kidney injury. These significant findings underscore the multifaceted role of CUR in modulating apoptosis and establish a foundation for its therapeutic applications in diverse disease contexts.

### Conclusions and perspectives

**Figure 1:**
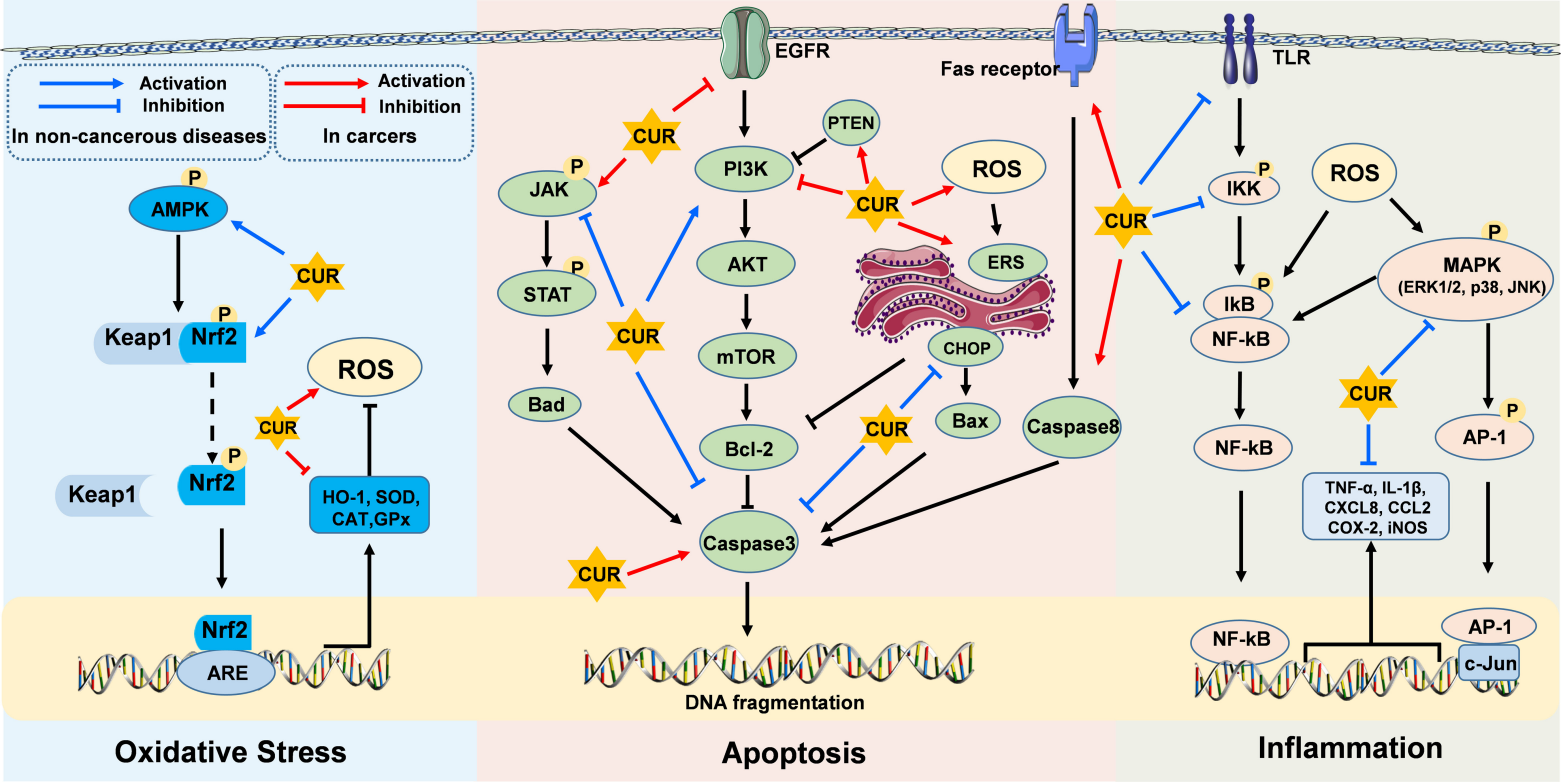
Molecular targets of curcumin on oxidative stress, inflammatory response, and apoptosis in non-cancerous diseases and cancers. CUR exerts its effects through diverse cellular core pathways, including the AMPK/Nrf2/ARE/Keap1 pathway associated with oxidative stress, the NF-κB/AP-1/MAPK pathways related to inflammatory responses, and the PI3K/AKT/mTOR, JAK/STAT, and ER stress-induced pathways involved in apoptotic mechanisms.

Curcumin, a bioactive compound present in turmeric, has emerged as a potential health-promoting agent in the regulation of various diseases. This mini-review offers a comprehensive summary of recent advances in elucidating the preventive and curative effects of CUR on oxidative stress, inflammation, and apoptosis in non-cancerous diseases and cancers. Generally, CUR exhibits its antioxidant property through AMPK/Nrf2/ARE/Keap1 pathway activation, its anti-inflammatory property via NF-κB/AP-1/MAPK pathways inhibition, and its anti-apoptosis property by blocking JAK/STAT and ER stress-induced pathways while activating PI3K/AKT/mTOR pathways in non-cancerous diseases (**Figure 1**). Conversely, CUR demonstrates pro-oxidant, anti-inflammatory, and pro-apoptosis properties in cancers (**Figure 1**). Therefore, the future perspectives of CUR’s application encompass exploring novel drug formulations, investigating combination therapy approaches, conducting disease-specific clinical trials, developing targeted therapies, and integrating them with lifestyle interventions to optimize its therapeutic potential in both non-cancerous diseases and cancers. However, further research is necessary to comprehensively elucidate the underlying mechanisms of CUR in alleviating host diseases and to devise more effective clinical strategies.

## Author contributions

DC and H-YL: conceptualization. PH, KL, X-XP, YK, T-JY, and Z-YW writing the original draft. H-YL, DC, and ZL: review and editing. All authors contributed to the article and approved the submitted version.
